# Modern Sociology

**Published:** 1902-07-12

**Authors:** 


### Modern Sociology.

### A NEGLECTED ASPECT OF THE EDUCATION BILL: BY A CORRESPONDENT.

There is one aspect of the Education Bill which, perhaps because of its non-party and non-political character, is in danger of being lost sight of—namely, the position of women with regard to the new authority. It was alluded to, briefly, by Mr. Acland, late Minister of Education, in a speech at Bristol on May 14th, but otherwise it has received but scant notice on public platforms. Mr. Acland said: "Now, in abolishing the School Boards, what are you doing? You are depriving the country of the services, the directly elected services, of some of the best educational enthusiasts in the country. You are depriving the country of the enormously useful work of many men, aye, of many women too, and the women can find no elected place on the new authorities, which is a grave disaster. . . . Some of them, no doubt, may be replaced, but only as nominees, and in doing that you are doing something which is the gravest injury to education."

Mr. Balfour's reply to the question, when raised in Parliament, is reported to have been as follows:—"That the fact that women were not specifically mentioned in the first clause of the Education Act was not done with a view to excluding women, but because there would be some confusion in the Bill unless the word were introduced throughout. The wording amply covered the case of women, and it was certainly contemplated that all women would bear their full share in the work of education."

The question is, What is a "person"? According to Locke, the definition is "a thinking, intelligent being," but English law does not bear him out, at least when the question is one of public office.

It will be remembered that when the voters in London proceeded to elect two women—Lady Sandhurst and Miss Cons—to the newly constituted County Council, their election was at once contested in the Law Courts and they were rejected. The judges held that "Whenever the subject-matter of an Act of Parliament is the right to the exercise of a public function, unless the contrary is expressly stated, the right to exercise that function is confined to men." So that Mr. Balfour's theory does not receive the support of legal expert opinion, and the definition of the word "persons," if disputed in the Law Courts, is likely to clinch the business on the wrong side. What is required, evidently, is an interpretation clause, definitely stating that the word "persons," wherever used, is to be taken to mean both men and women.

What is the position of co-opted "persons," in the technical sense, likely to be? Miss F. M. Marion Townsend, who has served in both capacities as elected and co-opted member for Bristol (the one on the School Board, the other on the Technical Instruction Committee of the City Council), said recently: "My position on the two bodies is quite different. On the School Board I am on an exact equality with every other member—man or woman. There is a sense of security about my position; I know that after each election I have three years of uninterrupted work before me. There is also a sense of responsibility which is most wholesome. The parents and ratepayers have a right to tell me what they consider best for the children; it is my duty to listen. If I endeavour to carry out their wishes, if they are satisfied with my work, I expect them in return to support me at the next election. A woman thus publicly elected, and thus responsible to the public, has a fair field for permanent, useful, solid work. On the Technical Instruction Committee I have not the sense of equal right, security, or responsibility that I have on the School Board. The co-option of outside members—*i.e.*, other than City Councillors—takes place once a year. If in advocating reforms or necessary alterations in methods of work I go against the opinions and prejudices of other members, I know that at the next yearly appointments such members can refuse to re-co-opt me. This insecurity of tenure, the sense of being no one's direct representative, and of being responsible to no one, makes it difficult to work with the same enthusiasm as when popularly elected. It takes the edge off the interest and is calculated to lessen one's permanent usefulness."

It should hardly be necessary to repeat the reasons why women should be represented on any public body that has to deal with children. Apart from the consideration that, as citizens, they should take their share in the administration of affairs, there are four obvious and cogent reasons. (1) The value of their influence, directly and indirectly, on the children, and the arrangements they may be able to make for their benefit. An instance occurred recently on a provincial School Board. A member (man) of the library sub-committee wanted to label the books in two lots, "For Boys," "For Girls," quite oblivious of the fact that books taken out by boys would be easily accessible to their sisters. One of the women members at once rose to the occasion. Why, she asked, should boys be allowed to read books of adventure, and girls be tied down to goody-goody stories written expressly for them? It was putting them on a lower intellectual plane at the very outset. She carried her point with the Board; and the very man who had made the proposal did not support it. It must be remembered that half the children in elementary schools are girls. (2) The enormous preponderance in the number of women teachers over men teachers. It may be said without exaggeration that the education of children in elementary schools is very largely in the hands of women. A return to the Board of Education, made in 1900, gives the figures as 80,057 women to 28,978 men, a majority of nearly two-thirds; and of girl pupil-teachers as 26,706, and boy pupil-teachers 6,643, so that in the immediate future the majority will be still more striking, only one-fourth being men. Headmasters are complaining that they cannot get boys to train, while the women have steadily increased since 1870, at which date they were slightly in a minority. It is worth while to notice, too, that ladies are entering this branch of the teaching profession by other means than the pupil-teacher course, but we do not hear of any men doing so. (3) The supervision of technical instruction in cooking, laundry-work, housekeeping, etc., generally acknowledged to be especially woman's work; and (4) The care and training of defective children. This is a branch of work which has made great advances in London, and that is being copied in some of the provincial towns. It was a woman who first drew attention to the fact that over-pressure was being used, and that classification was required. The boarding-out of children whose homes are too far from the special centre, is a development which is successful and increasing, and here there is great scope for women in supervising the local committees.

But there is a further consideration, summed up in the phrase *ad hoc*, for, as Lady Strachey has lately pointed out, "for educational authorities elected *ad hoc* it would probably be easier to find a sufficient number of suitable women willing to serve; for it is one thing to undertake a special branch of work and quite another to undertake along with it the multifarious duties falling to the lot of a member of a Town or County Council."

This to many people is the crux of the whole matter, but it affects men as well as women, and so lies outside the scope of this article.

